# Inactivation of Autophagy in Keratinocytes Reduces Tumor Growth in Mouse Models of Epithelial Skin Cancer

**DOI:** 10.3390/cells11223691

**Published:** 2022-11-21

**Authors:** Caterina Barresi, Heidemarie Rossiter, Maria Buchberger, Johannes Pammer, Supawadee Sukseree, Maria Sibilia, Erwin Tschachler, Leopold Eckhart

**Affiliations:** 1 Department of Dermatology, Medical University of Vienna, 1090 Vienna, Austria; 2 Clinical Institute of Pathology, Medical University of Vienna, 1090 Vienna, Austria; 3 Center for Cancer Research, Comprehensive Cancer Center, Medical University of Vienna, 1090 Vienna, Austria

**Keywords:** autophagy, keratinocytes, carcinogenesis, tumor, squamous cell carcinoma, epidermis, epidermal growth factor, epithelium

## Abstract

Autophagy is a ubiquitous degradation mechanism, which plays a critical role in cellular homeostasis. To test whether autophagy suppresses or supports the growth of tumors in the epidermis of the skin, we inactivated the essential autophagy gene *Atg7* specifically in the epidermal keratinocytes of mice (*Atg7^∆ep^*) and subjected such mutant mice and fully autophagy-competent mice to tumorigenesis. The lack of epithelial Atg7 did not prevent tumor formation in response to 7, 12-dimethylbenz(a)anthracene (DMBA) as the initiator and 12-O tetradecanoylphorbol-13-acetate (TPA) as the promoter of tumor growth. However, the number of tumors per mouse was reduced in mice with epithelial Atg7 deficiency. In the *K5-SOS EGFR^wa2/wa2^* mouse model, epithelial tumors were initiated by Son of sevenless (SOS) in response to wounding. Within 12 weeks after tumor initiation, 60% of the autophagy-competent *K5-SOS EGFR^wa2/wa2^* mice had tumors of 1 cm diameter and had to be sacrificed, whereas none of the *Atg7^∆ep^ K5-SOS EGFR^wa2/wa2^* mice formed tumors of this size. In summary, the deletion of *Atg7* reduced the growth of epithelial tumors in these two mouse models of skin cancer. Thus, our data show that the inhibition of autophagy limits the growth of epithelial skin tumors.

## 1. Introduction 

Autophagy is a mechanism for the removal of damaged cellular organelles, protein aggregates, and macromolecules and for the recycling of their molecular components for reuse by the cells [1]. The substrates of macroautophagy are sequestered in double-membraned autophagosomes, which fuse with lysosomes, forming autolysosomes, in which degradation by lysosomal enzymes proceeds. Subsequently, the degradation products are released into the cytoplasm and can re-enter the cellular metabolic pathways. The formation of autophagosomes is a tightly regulated process controlled by specific sets of *autophagy-related* (*Atg*) genes [1,2,3]. Autophagy can be blocked by the deletion of essential genes such as *Atg7*. The global deletion of *Atg7* leads to perinatal mortality [4], whereas mice with the cell-type specific conditional deletion of *Atg7* are viable [5].

The role of autophagy in the development and progression of tumors is complex and depends on the type of cancer [6,7,8,9]. Autophagy was found to suppress tumors by counteracting genomic instability and reducing cell growth [10]. In established tumors, autophagy promotes the survival of malignant cells by mitigating metabolic stress and by preventing cell death and the ensuing activation of antitumor defense [11]. A low expression of *Atg12* in head and neck squamous cell carcinoma is associated with reduced tumor tolerance to hypoxia and a better prognosis for patients [12]. The inactivation of the autophagic machinery in experimental animals has also yielded partially contradictory results: The tissue-specific deletion of *Atg7* in mice resulted in the spontaneous development of benign hepatic neoplasms [13], and the deletion of *Atg5* increased K-ras-mediated tumor incidence in the lung [14]. The suppression of autophagy in the pancreas increased the emergence of tumors in the context of activated K-ras (G12D) and inactive p53, and either increased or decreased the progression to malignancy, depending on the experimental model [15,16]. The deletion of *Atg7* in a model of K-ras(G12D)-driven non-small-cell lung cancer reduced tumor burden [17].

Cancers of the skin epithelium, such as squamous cell carcinoma (SCC), are highly prevalent in humans and have been extensively studied in mouse models [18,19]. In the two-stage chemical carcinogenesis protocol, tumors are initiated by the topical application of 7,12-dimethylbenz[a]anthracene (DMBA), which causes the mutation of the *Hras* gene [20], and promoted by the topical application of 12-O-tetradecanoylphorbol 13-acetate (TPA), which triggers an inflammatory microenvironment [21]. In another model, the transgenic expression of the dominant active Son of sevenless (SOS), an activator of Ras signaling, under the control of the *keratin 5* (*K5*) promoter in epidermal keratinocytes [22] causes SCC-like skin tumorigenesis. Tumor growth is greatly reduced or suppressed by the presence of the *waved* (*wa2*) mutation of the epidermal growth factor receptor (EGFR) [22], but it can be efficiently induced by mechanical irritation or wounding [23].

The roles of autophagy in the epidermis and in epithelial skin cancers are not fully understood at present. The suppression of autophagy caused changes in the differentiation and aging-associated features of epithelial skin cells [24,25,26,27,28,29,30,31,32,33,34,35]. The skin barrier function of mice without epidermal autophagy was intact under non-stressed conditions [24]. However, the deletion of *Atg7* impaired the resistance of epidermal keratinocytes to intrinsic and environmental oxidative stress [36] and sensitized keratinocytes to apoptosis [37] and stress-induced senescence [36]. Autophagy is active in the normal epidermis [24,38,39], and its rate is enhanced in human squamous cell carcinomas [40].

**Figure 1:**
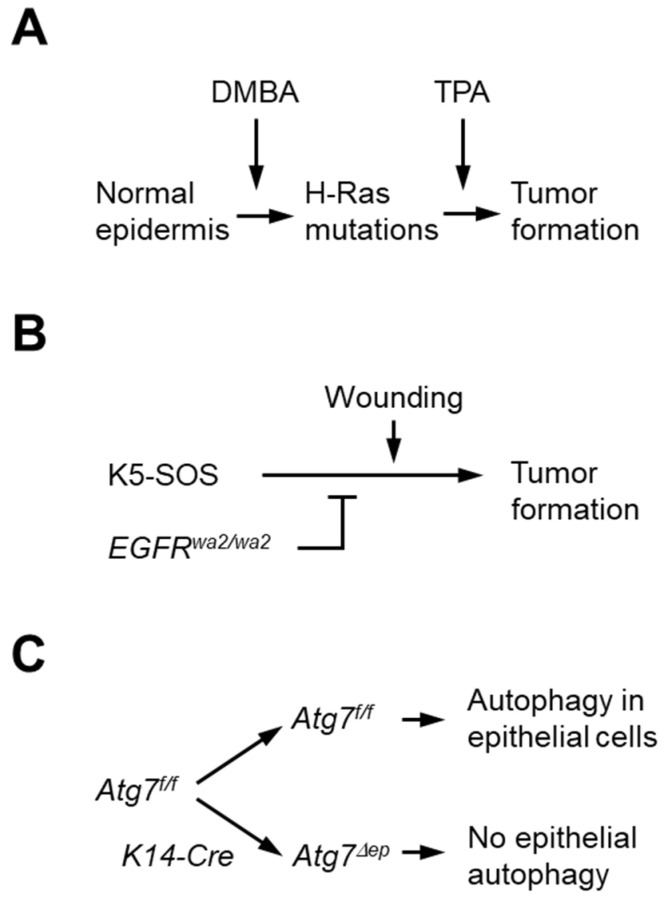
Models for testing the role of Atg7 in tumorigenesis. (**A**) In the two-stage carcinogenesis model, mice were treated with DMBA to initiate tumors through mutations in the oncogene H-Ras. Treatment with TPA in the following 25 weeks promoted tumor growth, which was monitored up to 50 weeks after DMBA treatment. (**B**) In the *K5-SOS EGFR^wa2/wa2^* model, the *SOS* transgene was expressed in keratinocytes to induce tumors. The waved mutation in EGFR suppressed tumor growth unless it was triggered by wounding of ear skin. Tumors were monitored up to 12 weeks after wounding. (**C**) To determine the role of Atg7-dependent autophagy, an essential segment of the *Atg7* gene was flanked by loxP sites (*Atg7^f/f^*, *Atg7* floxed), allowing expression of wild-type (WT) ATG7 protein in the absence of Cre recombinase, whereas expression of Cre under the control of the *keratin 14* (*K14*) promoter led to deletion of floxed Atg7 and blockade of autophagy in epithelial cells of the skin of *K14-Cre Atg7^f/f^* (also referred to as *Atg7^Δep^*) mice.

Here, we investigated the role of autophagy in the initiation and progression of epithelial skin tumors by ablating *Atg7* in two mouse models of skin cancer (Figure 1 [22]): DMBA/TPA-mediated two-stage carcinogenesis and genetically driven carcinogenesis in the *K5-SOS EGFR^wa2/wa2^* model [22]. The epidermal keratinocyte-targeted deletion of *Atg7* (*Atg7^Δ^^ep^*) was achieved by the Cre-recombinase-mediated deletion of an essential *Atg7* gene segment flanked by loxP sites (*Atg7^f/f^*), as reported previously [24]. The abrogation of *Atg7* diminished tumor growth to different extents in these two models of skin carcinogenesis.

## 2. Materials and Methods

### 2.1. Animals

The generation of *K14-Cre Atg7^f/f^* (*Atg7^∆ep^*) mice and the absence of aberrant inflammation in the skin and other organs, as well as the normal body mass of these mice, have been previously reported [24,25]. Tumors were induced in these mice by using the classical two-stage chemical carcinogenesis model (see below). To provide a second tumor model, *Atg7^∆ep^* mice and the corresponding *K14-Cre*-negative *Atg7^f/f^* controls were crossed to *K5-SOS EGFR^wa2/wa2^* mice [22], to yield mice homozygous for *EGFR^wa2/wa2^* and *Atg7^f/f^* and hemizygous for *K5-SOS* and the *K14-Cre* transgene. *K5-SOS EGFR^wa2/wa2^* mice develop tumors spontaneously at low frequency, and the incidence can be increased through wounding [22]. The mice were kept on a normal cycle of 12 h alternating light and dark and allowed access to fodder and water ad libitum. Animal experiments were conducted according to the guidelines of the Declaration of Helsinki and approved by the Ethics Review Committee for Animal Experimentation of the Medical University of Vienna, Austria, and the Bundesministerium für Wissenschaft und Forschung, Austria (protocol: GZ 66.009/0120-II10b/2010, 02.04.2010).

### 2.2. Chemical Carcinogenesis Model

Chemical carcinogenesis was performed in *K14-Cre Atg7^f/f^* females (*n* = 17, 8–12 weeks old) and control *Atg7^f/f^* females (*n* = 20, 8–12 weeks old). The mice were shaved on their backs with electric clippers 1 day before the beginning of the treatment and received a single application (50 μL of a 100 nM solution) of DMBA (Sigma Aldrich, Vienna, Austria). One week later, all the mice were treated with three applications of TPA (6 nM, Sigma Aldrich, Vienna, Austria) in acetone (volume of 50 μL per application) per week for 25 weeks. All the mice were monitored for tumor development once a week for 50 weeks, and tumors >1 mm were recorded. Tumor size was measured with calipers in 2 dimensions, and the mean of the two was defined as the tumor size. One *K14-Cre Atg7^f/f^* mouse died without a tumor in week 20 of TPA treatment and was not included in the statistical analysis. Downward-invading tumors were recorded as carcinomas upon histological confirmation. The mice with tumors of a diameter >10 mm were euthanized. All the animals underwent postmortem examination for the presence of non-epidermal tumors, which were not found in any animal.

### 2.3. K5-SOS Carcinogenesis Model

For tumor initiation in the *K5-SOS EGFR^wa2/wa2^ Atg7^f/f^* mouse line, a mouse ear punch with a 2 mm diameter was used to place small wounds on the ear tips of 7–10-week-old female mice. The animals were monitored for tumor formation once weekly and were euthanized via CO_2_ asphyxiation either when tumors reached a diameter of 10 mm or at 12 weeks after initiation. Tumor volume was calculated using the formula V = L × W × W × pi/6 [41]. 

### 2.4. Statistical Analysis

GraphPad Prism version 8.0.1 for Windows, GraphPad Software, San Diego, California, USA, was used to analyze the data. Tumors per mouse and the size of tumors in *Atg7^f/f^* and *Atg7*^*Δ*^*^ep^* mice were compared with a two-tailed, unpaired Student’s *t*-test. All data are presented as mean ± standard error of the mean. * *p*-values < 0.05; ** *p*-values < 0.01. Survival curves were analyzed with the Log-rank (Mantel–Cox) test and the Gehan–Breslow–Wilcoxon test.

## 3. Results

### 3.1. Inactivation of Atg7 in Keratinocytes Does Not Alter the Incidence of Chemically Induced Skin Tumors but Leads to Lower Tumor Numbers per Mouse 

**Figure 2:**
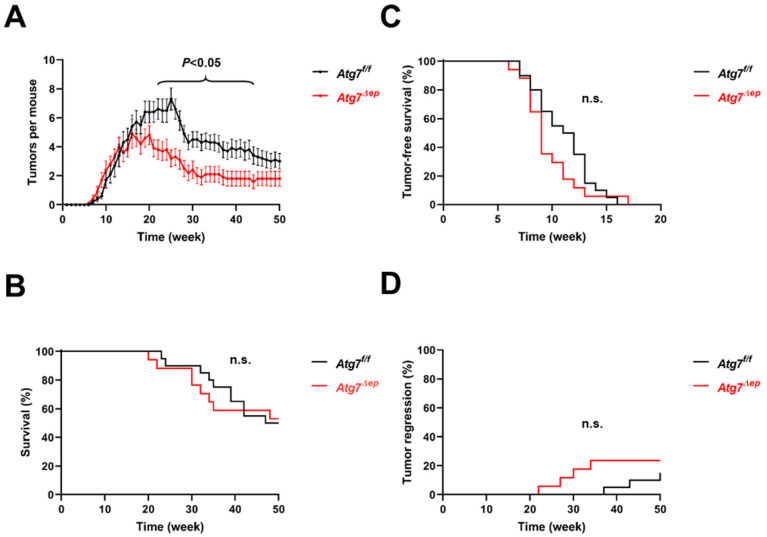
Deletion of *Atg7* leads to lower numbers of tumors per mouse in the two-step chemical carcinogenesis model. (**A**) *Atg7^f/f^* (*n* = 20) and *Atg7^Δep^* (*n* = 17) mice were treated with DMBA in week 0 and with TPA in the subsequent 25 weeks. The mean number of tumors per mouse is plotted over time. Error bars indicate standard error of the mean (SEM). For mice that died, the final tumor number before death was kept until week 50. The difference between *Atg7^f/f^* and *Atg7^Δep^* was significant (*p* < 0.05, unpaired *t*-test) in weeks 21–44. *p* < 0.01 in weeks 22, 25–29, and 31–35. (**B**) Survival of mice is shown in a Kaplan–Meier plot. Differences were not significant (n.s.). (**C**) Tumor-free survival of mice is shown in a Kaplan–Meier plot. Differences were not significant. (**D**) Tumor regression was defined as the absence of tumors until week 50 in mice that previously had at least one tumor. The percentage of mice in which tumors regressed is shown over time. Differences were not significant.

To determine the role of autophagy in skin tumor formation, we first subjected epidermis-specific *Atg7* knockout mice (*Atg7^Δep^*) and control littermates (*Atg7^f/f^*) to the DMBA/TPA protocol of skin carcinogenesis [19]. *Atg7^Δep^* and *Atg7^f/f^* mice developed papillomas at a similar rate, but the tumor numbers per animal were lower in *Atg7^Δep^* than in *Atg7^f/f^* mice between weeks 21 and 44 (Figure 2A; Table S1). The maximum number of tumors per mouse was 4.9 in *Atg7^Δep^* mice at week 21 and 7.3 in *Atg7^f/f^* mice at week 25 (Figure 2A). The mean tumor load decreased after the end of TPA treatment at week 25. Survival (Figure 2B) and tumor-free survival (Figure 2C) did not significantly differ between *Atg7^Δep^* and *Atg7^f/f^* mice. After all the mice developed tumors (with the exception of one *Atg7^Δep^* mouse which had to be excluded from the analysis; see Methods for details), 4 out of 17 *Atg7^Δep^* and 3 out of 20 *Atg7^f/f^* mice showed tumor regression at the end of the study (week 50), with the time course of regression not significantly different between the two genotypes (Figure 2D). The conversion of papilloma into carcinomas was observed in 11.1% of the *Atg7^Δep^* and 7.7% of the *Atg7^f/f^* mice (data not shown). These data suggested that the absence of autophagy was compatible with tumor formation but not with the maximal growth of epithelial skin tumors, though the overall survival was not improved in this experiment.

### 3.2. Epidermal Keratinocyte-Specific Atg7 Deletion Impairs K5-SOS Dependent Tumor Growth

**Figure 3:**
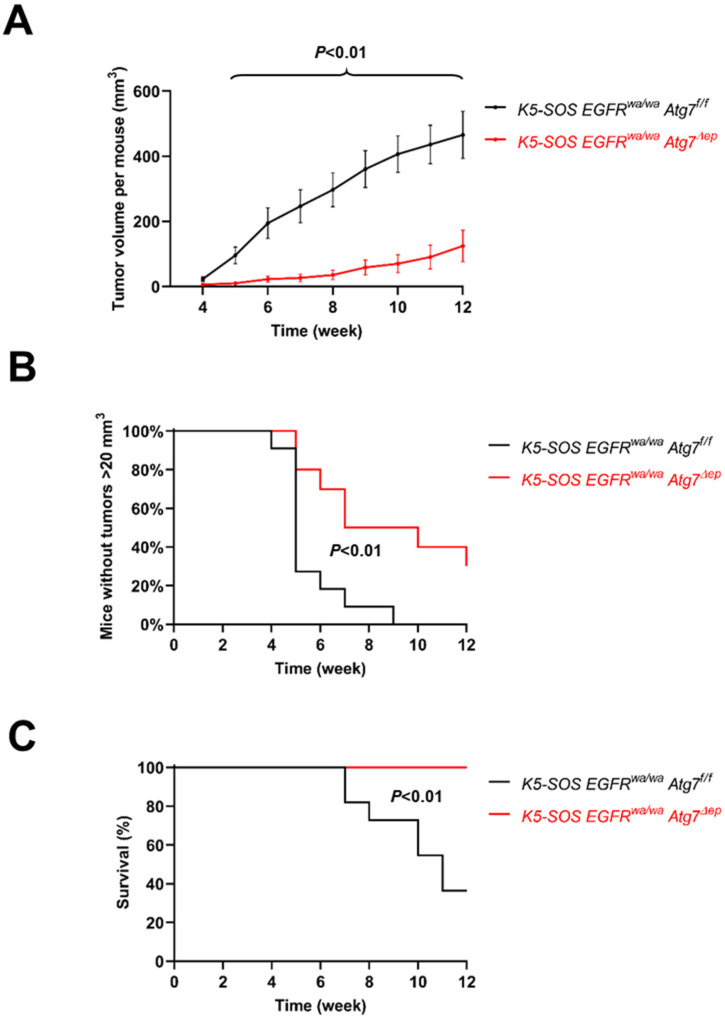
Inactivation of *Atg7* impairs *K5-SOS*-dependent skin tumor formation. (**A**) *K5-SOS EGFR^wa2/wa2^ Atg7^f/f^* (*n* = 11) and *K5-SOS EGFR^wa2/wa2^ Atg7^Δep^* (*n* = 10) mice were wounded on both ears in week 0 and monitored over the next 12 weeks. The tumor volume was determined every week. Error bars indicate standard error of the mean (SEM). For mice that had to be killed because the tumor diameter reached 10 mm or more, the final tumor volume before death was kept until week 12. The difference between *Atg7^f/f^* and *Atg7^Δep^* was significant with *p* < 0.05 (unpaired *t*-test) at week 4 and with *p* < 0.01 in weeks 2–12. (**B**) The absence of tumors with a volume > 20 mm^3^ is plotted over time. The difference between *Atg7^f/f^* and *Atg7^Δep^* was significant (*p* < 0.01, Log-rank (Mantel–Cox) test and Gehan–Breslow–Wilcoxon test). (**C**) Survival of mice. Mice were killed when the diameter of a tumor reached 10 mm. The difference between *Atg7^f/f^* and *Atg7^Δep^* was significant (*p* < 0.01, Log-rank (Mantel–Cox) test and Gehan–Breslow–Wilcoxon test).

We next investigated the development of tumors in the *K5-SOS EGFR^wa2/wa2^* mouse model [22,23]. In these mice, tumors can be induced by the wounding of the skin. The *K5-SOS* transgene and the *EGFR^wa2^* allele were crossed into *Atg7^Δep^* and *Atg7^f/f^* mice to generate *K5-SOS EGFR^wa2/wa2^ Atg7^Δep^* and *K5-SOS EGFR^wa2/wa2^ Atg7^f/f^* mice. The ear tips of 7–10-week-old female mice of these two genotypes were wounded with an ear punch. Tumors became macroscopically visible at about 4 weeks after wounding in both groups of mice, with 11 out of 11 *K5-SOS EGFR^wa2/wa2^ Atg7^f/f^* and 8 out 10 of *K5-SOS EGFR^wa2/wa2^ Atg7^Δep^* mice developing tumors (Table S2). The mean tumor volume was significantly larger in the fully autophagy-competent mice (*K5-SOS EGFR^wa2/wa2^ Atg7^f/f^*) than in mice lacking Atg7 in epithelial cells (*K5-SOS EGFR^wa2/wa2^ Atg7^Δep^*) from week 4 until week 12 (Figure 3A). Tumors with a volume larger than 20 mm³ on one or both ears, a threshold defined in a previous study [23], appeared in 11 out of 11 *K5-SOS EGFR^wa2/wa2^ Atg7^f/f^* and 7 out of 10 *K5-SOS EGFR^wa2/wa2^ Atg7^Δep^* mice (Table S3). The corresponding tumor-size-survival curves were significantly different between the two genotypes (Figure 3B). Tumors of 10 mm diameter formed in 6 out of the 11 *K5-SOS EGFR^wa2/wa2^ Atg7^f/f^* mice, which subsequently had to be killed, but did not form in any of the 10 *K5-SOS EGFR^wa2/wa2^ Atg7^Δep^* mice, with the difference being statistically significant (Figure 3C). Collectively, the deletion of *Atg7* reduced the growth of tumors more efficiently in the genetically controlled *K5-SOS EGFR^wa2/wa2^* model than in DMBA/TPA-dependent chemical carcinogenesis.

## 4. Discussion

Autophagy is a tightly regulated degradation process that is implicated in the homeostasis of normal, stressed, and malignantly transformed cells. Alterations in autophagic activity (autophagic flux) have been shown to have beneficial or detrimental effects on the health and survival of organisms depending on the specific tissue context in which autophagy was targeted [3]. In the present study, autophagy was blocked in the epithelial skin cells of two mouse models of tumorigenesis (Figure 1). The absence of Atg7 from epithelial cells was associated with lower tumor numbers per mouse in response to chemical carcinogenesis (Figure 2) and with reduced tumor size and lower mortality in carcinogenesis driven by the activation of Ras-dependent signaling through transgenic SOS (Figure 3). These results suggest that Atg7-dependent autophagy supports tumor growth and the deletion of *Atg7* reduces tumor growth. 

The lack of autophagy was associated with fewer tumors after chemical carcinogenesis. This difference was caused by enhanced tumor regression in the *Atg7^Δep^* mice, compared with the wild-type mice. By contrast, the rates of tumorigenesis and survival did not significantly differ between the *Atg7^Δep^* and the control mice. It is thus likely that autophagy supports the persistence of tumors in this model and, accordingly, the suppression of autophagy decreases the tumor load of mice. Previous studies showed that the deletion of *Atg7* impaired the resistance of epidermal keratinocytes to intrinsic and environmental oxidative stress [36] and sensitized keratinocytes to apoptosis [37] and stress-induced senescence [36]. The lack of autophagy-dependent protection against stress at the cellular level may also reduce the growth and survival of malignant keratinocytes in our model. Therefore, in future studies, it will be interesting to test whether the potential tumor-suppressive effect of the inhibition of autophagy is more pronounced if malignant epithelial cells are exposed to increased levels of stress, such as irradiation or persistent exposure to toxic chemicals. 

In the *K5-SOS EGFR^wa2/wa2^* model of skin cancer, the inactivation of *Atg7* was compatible with tumorigenesis in 8 out of 10 mice, but 2 mice did not develop tumors. By contrast, all the fully autophagy-competent mice developed tumors. This result suggests that inactivating autophagy has a minor protective effect against carcinogenesis in this model. The role of autophagy in the response to skin wounding [42] may affect wounding-induced tumorigenesis in this model. More importantly, however, the development of large tumors was considerably and significantly reduced by blocking autophagy, and none of the *K5-SOS EGFR^wa2/wa2^ Atg7^Δep^* mice developed tumors of a diameter of 10 mm or more. This led to higher survival of *K5-SOS EGFR^wa2/wa2^ Atg7^Δep^* since none of these animals had to be sacrificed. These results point to an important role of autophagy in supporting tumor growth. Accordingly, the inhibition of autophagy had an antitumor effect, which, however, was less clear than the abrogation of vascular endothelial growth factor (VEGF). When VEGF was deleted in the keratinocytes of *K5-SOS EGFR^wa2/wa2^* mice, the formation of large tumors was completely abrogated and all the *K5-SOS EGFR^wa2/wa2^ VEGF^Δep^* mice survived [23]. Therefore, the beneficial effect of suppressing autophagy is likely smaller than that achievable with other treatments such as the blockade of VEGF. As discussed above, the inhibition of autophagy may be considered an adjuvant therapy but not a stand-alone therapy for skin tumors.

The present study was designed to test the antitumor effect of blocking autophagy in all epithelial cells, but we did not dissect the effects of autophagy in tumor cells and in the surrounding epithelium. Although the abrogation of the cell-autonomous functions of autophagy in tumor cells may suffice to cause the beneficial effects, additional effects involving changes in non-malignant cells are possible. Previous studies suggested that the suppression of epithelial autophagy impairs DNA damage recognition and nucleotide excision repair [43], which may support tumorigenesis, whereas protumorigenic inflammatory factors in the cutaneous microenvironment were reduced [37]. In contrast to a report on the protective effects of *Atg7* deletion in UV-dependent carcinogenesis [37], the deletion of *Atg7* did not significantly alter the rate of tumor formation in our models. We observed an antitumor effect of the inhibition of autophagy when tumor growth was monitored over a longer time. The tumor cells’ dependency on autophagy for persistent growth is well-explained by the central function of autophagy as a key component of intracellular recycling. Various models have suggested that autophagy increases the fitness of neoplastic cells, in particular when the supply of nutrients decreases in large tumors [44,45,46]. However, autophagy in the cells of the tumor microenvironment also supports the growth of tumors by enhancing the generation and secretion of nutrients [47,48] and the autophagy-dependent signaling processes in non-malignant cells affect their interactions with tumor cells [49,50]. Thus, the underlying mechanisms through which the inhibition of autophagy reduces the growth of skin tumors and the antitumor efficacy of targeting autophagy alone or in combination with other therapies remain to be determined. 

## Data Availability

Data are contained within the article or Supplementary Materials.

## Supplementary Materials

The following are available online at https://www.mdpi.com/article/10.3390/cells11223691/s1, Table S1: Tumor numbers in DMBA/TPA-treated *Atg7^f/f^* and *Atg7^Δep^* mice. Table S2: Tumor volume of *K5-SOS EGFR^wa2/wa2^ Atg7^f/f^* and *K5-SOS EGFR^wa2/wa2^ Atg7^Δep^* mice after wounding on both ears, including volumes after death of affected mice. Table S3: Tumor volume on each ear of *K5-SOS EGFR^wa2/wa2^ Atg7^f/f^* and *K5-SOS EGFR^wa2/wa2^ Atg7^Δep^* mice.

## References

[1] Mizushima N., Komatsu M. (2011). Autophagy: Renovation of cells and tissues. Cell.

[2] Mizushima N. (2020). The ATG conjugation systems in autophagy. Curr. Opin. Cell Biol..

[3] Klionsky D.J., Petroni G., Amaravadi R.K., Baehrecke E.H., Ballabio A., Boya P., Bravo-San Pedro J.M., Cadwell K., Cecconi F., Choi A.M.K. (2021). Autophagy in major human diseases. EMBO J..

[4] Komatsu M., Waguri S., Ueno T., Iwata J., Murata S., Tanida I., Ezaki J., Mizushima N., Ohsumi Y., Uchiyama Y. (2005). Impairment of starvation-induced and constitutive autophagy in Atg7-deficient mice. J. Cell Biol..

[5] Kuma A., Komatsu M., Mizushima N. (2017). Autophagy-monitoring and autophagy-deficient mice. Autophagy.

[6] Galluzzi L., Pietrocola F., Bravo-San Pedro J.M., Amaravadi R.K., Baehrecke E.H., Cecconi F., Codogno P., Debnath J., Gewirtz D.A., Karantza V. (2015). Autophagy in malignant transformation and cancer progression. EMBO J..

[7] White E. (2015). The role for autophagy in cancer. J. Clin. Investig..

[8] Hernandez G.A., Perera R.M. (2022). Autophagy in cancer cell remodeling and quality control. Mol. Cell.

[9] Russell R.C., Guan K.L. (2022). The multifaceted role of autophagy in cancer. EMBO J..

[10] Mathew R., Karp C.M., Beaudoin B., Vuong N., Chen G., Chen H.Y., Bray K., Reddy A., Bhanot G., Gelinas C. (2009). Autophagy suppresses tumorigenesis through elimination of p62. Cell.

[11] Degenhardt K., Mathew R., Beaudoin B., Bray K., Anderson D., Chen G., Mukherjee C., Shi Y., Gélinas C., Fan Y. (2006). Autophagy promotes tumor cell survival and restricts necrosis, inflammation, and tumorigenesis. Cancer Cell.

[12] Keulers T.G., Koch A., van Gisbergen M.W., Barbeau L.M.O., Zonneveld M.I., de Jong M.C., Savelkouls K.G.M., Wanders R.G., Bussink J., Melotte V. (2022). ATG12 deficiency results in intracellular glutamine depletion, abrogation of tumor hypoxia and a favorable prognosis in cancer. Autophagy.

[13] Takamura A., Komatsu M., Hara T., Sakamoto A., Kishi C., Waguri S., Eishi Y., Hino O., Tanaka K., Mizushima N. (2011). Autophagy-deficient mice develop multiple liver tumors. Genes Dev..

[14] Rao S., Tortola L., Perlot T., Wirnsberger G., Novatchkova M., Nitsch R., Sykacek P., Frank L., Schramek D., Komnenovic V. (2014). A dual role for autophagy in a murine model of lung cancer. Nat. Commun..

[15] Rosenfeldt M.T., O’Prey J., Morton J.P., Nixon C., MacKay G., Mrowinska A., Au A., Rai T.S., Zheng L., Ridgway R. (2013). p53 status determines the role of autophagy in pancreatic tumour development. Nature.

[16] Yang A., Rajeshkumar N.V., Wang X., Yabuuchi S., Alexander B.M., Chu G.C., Von Hoff D.D., Maitra A., Kimmelman A.C. (2014). Autophagy is critical for pancreatic tumor growth and progression in tumors with p53 alterations. Cancer Discov..

[17] Guo J.Y., Karsli-Uzunbas G., Mathew R., Aisner S.C., Kamphorst J.J., Strohecker A.M., Chen G., Price S., Lu W., Teng X. (2013). Autophagy suppresses progression of K-ras-induced lung tumors to oncocytomas and maintains lipid homeostasis. Genes Dev..

[18] Hanahan D., Wagner E.F., Palmiter R.D. (2007). The origins of oncomice: A history of the first transgenic mice genetically engineered to develop cancer. Genes Dev..

[19] Balmain A., Yuspa S.H. (2014). Milestones in skin carcinogenesis: The biology of multistage carcinogenesis. J. Investig. Dermatol..

[20] Quintanilla M., Brown K., Ramsden M., Balmain A. (1986). Carcinogen-specific mutation and amplification of Ha-ras during mouse skin carcinogenesis. Nature.

[21] Mueller M.M. (2006). Inflammation in epithelial skin tumours: Old stories and new ideas. Eur. J. Cancer.

[22] Sibilia M., Fleischmann A., Behrens A., Stingl L., Carroll J., Watt F.M., Schlessinger J., Wagner E.F. (2000). The EGF receptor provides an essential survival signal for SOS-dependent skin tumor development. Cell.

[23] Lichtenberger B.M., Tan P.K., Niederleithner H., Ferrara N., Petzelbauer P., Sibilia M. (2010). Autocrine VEGF signaling synergizes with EGFR in tumor cells to promote epithelial cancer development. Cell.

[24] Rossiter H., König U., Barresi C., Buchberger M., Ghannadan M., Zhang C.-F., Mlitz V., Gmeiner R., Sukseree S., Födinger D. (2013). Epidermal keratinocytes form a functional skin barrier in the absence of Atg7 dependent autophagy. J. Dermatol. Sci..

[25] Sukseree S., Mildner M., Rossiter H., Pammer J., Zhang C.F., Watanapokasin R., Tschachler E., Eckhart L. (2012). Autophagy in the thymic epithelium is dispensable for the development of self-tolerance in a novel mouse model. PLoS ONE.

[26] Sukseree S., Rossiter H., Mildner M., Pammer J., Buchberger M., Gruber F., Watanapokasin R., Tschachler E., Eckhart L. (2013). Targeted deletion of Atg5 reveals differential roles of autophagy in keratin K5-expressing epithelia. Biochem. Biophys. Res. Commun..

[27] Akinduro O., Sully K., Patel A., Robinson D.J., Chikh A., McPhail G., Braun K.M., Philpott M.P., Harwood C.A., Byrne C. (2016). Constitutive autophagy and nucleophagy during epidermal differentiation. J. Investig. Dermatol..

[28] Rossiter H., Stübiger G., Gröger M., König U., Gruber F., Sukseree S., Mlitz V., Buchberger M., Oskolkova O., Bochkov V. (2018). Inactivation of autophagy leads to changes in sebaceous gland morphology and function. Exp. Dermatol..

[29] Sukseree S., Bergmann S., Pajdzik K., Sipos W., Gruber F., Tschachler E., Eckhart L. (2018). Suppression of epithelial autophagy compromises the homeostasis of sweat glands during aging. J. Investig. Dermatol..

[30] Sukseree S., Bergmann S., Pajdzik K., Tschachler E., Eckhart L. (2018). Suppression of autophagy perturbs turnover of sequestosome-1/p62 in Merkel cells but not in keratinocytes. J. Dermatol. Sci..

[31] Cau L., Takahara H., Thompson P.R., Serre G., Méchin M.C., Simon M. (2019). Peptidylarginine deiminase inhibitor Cl-amidine attenuates cornification and interferes with the regulation of autophagy in reconstructed human epidermis. J. Investig. Dermatol..

[32] Jaeger K., Sukseree S., Zhong S., Phinney B.S., Mlitz V., Buchberger M., Narzt M.S., Gruber F., Tschachler E., Rice R.H. (2019). Cornification of nail keratinocytes requires autophagy for bulk degradation of intracellular proteins while sparing components of the cytoskeleton. Apoptosis.

[33] Eckhart L., Tschachler E., Gruber F. (2019). Autophagic control of skin aging. Front. Cell Dev. Biol..

[34] Simpson C.L., Tokito M.K., Uppala R., Sarkar M.K., Gudjonsson J.E., Holzbaur E.L.F. (2021). NIX initiates mitochondrial fragmentation via DRP1 to drive epidermal differentiation. Cell Rep..

[35] Liu C., Gu L., Ding J., Meng Q., Li N., Dai G., Li Q., Wu X. (2021). Autophagy in skin barrier and immune-related skin diseases. J. Dermatol..

[36] Song X., Narzt M.S., Nagelreiter I.M., Hohensinner P., Terlecki-Zaniewicz L., Tschachler E., Grillari J., Gruber F. (2017). Autophagy deficient keratinocytes display increased DNA damage, senescence and aberrant lipid composition after oxidative stress in vitro and in vivo. Redox Biol..

[37] Qiang L., Sample A., Shea C.R., Soltani K., Macleod K.F., He Y.Y. (2017). Autophagy gene ATG7 regulates ultraviolet radiation-induced inflammation and skin tumorigenesis. Autophagy.

[38] Yoshihara N., Ueno T., Takagi A., Oliva Trejo J.A., Haruna K., Suga Y., Komatsu M., Tanaka K., Ikeda S. (2015). The significant role of autophagy in the granular layer in normal skin differentiation and hair growth. Arch. Dermatol. Res..

[39] Wang Z., Zhou H., Zheng H., Zhou X., Shen G., Teng X., Liu X., Zhang J., Wei X., Hu Z. (2021). Autophagy-based unconventional secretion of HMGB1 by keratinocytes plays a pivotal role in psoriatic skin inflammation. Autophagy.

[40] Qiang L., Wu C., Ming M., Viollet B., He Y.Y. (2013). Autophagy controls p38 activation to promote cell survival under genotoxic stress. J. Biol. Chem..

[41] Tomayko M.M., Reynolds C.P. (1989). Determination of subcutaneous tumor size in athymic (nude) mice. Cancer Chemother. Pharmacol..

[42] Qiang L., Yang S., Cui Y.H., He Y.Y. (2021). Keratinocyte autophagy enables the activation of keratinocytes and fibroblasts and facilitates wound healing. Autophagy.

[43] Qiang L., Zhao B., Shah P., Sample A., Yang S., He Y.Y. (2016). Autophagy positively regulates DNA damage recognition by nucleotide excision repair. Autophagy.

[44] Rybstein M.D., Bravo-San Pedro J.M., Kroemer G., Galluzzi L. (2018). The autophagic network and cancer. Nat. Cell Biol..

[45] Lim J., Murthy A. (2020). Targeting autophagy to treat cancer: Challenges and opportunities. Front. Pharmacol..

[46] Patergnani S., Missiroli S., Morciano G., Perrone M., Mantovani C.M., Anania G., Fiorica F., Pinton P., Giorgi C. (2021). Understanding the role of autophagy in cancer formation and progression is a real opportunity to treat and cure human cancers. Cancers.

[47] Sousa C.M., Biancur D.E., Wang X., Halbrook C.J., Sherman M.H., Zhang L., Kremer D., Hwang R.F., Witkiewicz A.K., Ying H. (2016). Pancreatic stellate cells support tumour metabolism through autophagic alanine secretion. Nature.

[48] Katheder N.S., Khezri R., O’Farrell F., Schultz S.W., Jain A., Rahman M.M., Schink K.O., Theodossiou T.A., Johansen T., Juhász G. (2017). Microenvironmental autophagy promotes tumour growth. Nature.

[49] Cadwell K. (2016). Crosstalk between autophagy and inflammatory signalling pathways: Balancing defence and homeostasis. Nat. Rev. Immunol..

[50] Xia H., Green D.R., Zou W. (2021). Autophagy in tumour immunity and therapy. Nat. Rev. Cancer..

