# Supplementary material for: Inactivation of Autophagy in Keratinocytes Reduces Tumor Growth in Mouse Models of Epithelial Skin Cancer
Source: Cells. 2022 Nov 21;11(22):3691. doi: 10.3390/cells11223691 (PMC9688105; doi:10.3390/cells11223691)
Supplement: Supplementary file 1 [file cells-11-03691-s001.zip › Supplement.pdf]

Table S1. Tumor numbers in DMBA/TPA-treated *Atg7*$^{f/f}$ and *Atg7*$^{\Delta ep}$ mice

Mouse	Genotype	Number of tumors at different times after DMBA treatment (w, week)																																																	
		w1	w2	w3	w4	w5	w6	w7	w8	w9	w10	w11	w12	w13	w14	w15	w16	w17	w18	w19	w20	w21	w22	w23	w24	w25	w26	w27	w28	w29	w30	w31	w32	w33	w34	w35	w36	w37	w38	w39	w40	w41	w42	w43	w44	w45	w46	w47	w48	w49	w50
WT1	$Atg7^{f/f}$	0	0	0	0	0	0	0	0	0	0	0	0	0	0	0	1	2	3	4	5	6	5	2	4	4	5	6	5	5	5	4	4	4	4	3	2	3	1	1	1	1	2	2	0	0	0	0	0	0	0
WT2	$Atg7^{f/f}$	0	0	0	0	0	0	0	0	0	0	0	0	3	5	5	5	6	6	6	6	7	6	5	4	4	4	4	2	1	1	1	1	1	1	1	1	1	1	1	1	1	1	1	1	1	1	1	1	1	1
WT3	$Atg7^{f/f}$	0	0	0	0	0	0	0	0	0	0	0	0	1	1	4	4	5	5	5	6	5	6	6	6	7	7	7	6	6	6	6	5	4	4	6	6	5	5	5	7	7	5	5	5	3	3	2	2	2	3
WT4	$Atg7^{f/f}$	0	0	0	0	0	0	0	0	1	1	1	1	3	3	4	5	9	8	7	8	5	5	5	5	5	5	5	5	5	5	5	5	5	5	5	5	5	5	5	5	5	5	5	5	5	5	5	5	5	5
WT5	$Atg7^{f/f}$	0	0	0	0	0	0	0	0	0	0	0	0	0	0	1	1	1	4	7	2	3	2	2	3	9	10	7	6	8	9	7	7	8	7	10	7	7	7	9	8	7	7	10	8	7	7	6	6	6	6
WT6	$Atg7^{f/f}$	0	0	0	0	0	0	0	0	0	2	6	7	8	10	12	10	11	12	13	13	11	12	12	5	5	3	2	2	1	1	1	0	1	1	1	1	0	0	0	0	0	0	0	0	0	0	0	0	0	0
WT7	$Atg7^{f/f}$	0	0	0	0	0	0	0	1	1	7	7	13	13	13	13	14	15	9	12	11	14	14	12	12	14	12	11	9	3	5	3	3	4	2	2	3	3	3	2	2	2	1	1	1	1	1	1	1	1	1
WT8	$Atg7^{f/f}$	0	0	0	0	0	0	0	0	0	0	3	4	4	5	6	11	7	8	11	13	12	11	14	14	14	11	6	4	4	5	6	7	7	6	6	6	6	6	6	5	4	4	4	4	4	4	4	4	4	4
WT9	$Atg7^{f/f}$	0	0	0	0	0	0	0	1	1	1	2	4	5	7	9	10	8	4	5	3	7	7	7	8	10	9	10	8	6	6	7	5	7	6	6	6	6	6	6	6	6	6	6	6	6	6	6	6	6	6
WT10	$Atg7^{f/f}$	0	0	0	0	0	0	0	0	0	0	0	0	0	2	2	2	2	4	4	4	4	5	5	5	6	5	4	2	2	2	2	2	2	2	2	2	2	2	2	2	2	2	2	2	2	2	1	1	1	1
WT11	$Atg7^{f/f}$	0	0	0	0	0	0	0	0	0	0	0	1	1	3	1	2	2	2	2	3	3	4	4	4	7	7	8	7	5	5	7	6	5	6	6	6	4	4	5	4	5	4	5	2	3	2	3	4	4	4
WT12	$Atg7^{f/f}$	0	0	0	0	0	0	0	0	0	0	0	1	1	2	2	2	2	2	2	4	4	2	2	2	1	1	1	1	1	2	2	2	2	2	1	0	0	0	0	1	1	1	0	0	0	0	0	0	0	0
WT13	$Atg7^{f/f}$	0	0	0	0	0	0	0	0	0	0	0	0	2	2	2	3	3	3	6	3	3	3	1	0	5	1	1	2	2	2	2	2	3	3	2	2	2	2	2	2	2	2	2	2	2	2	1	1	1	0
WT14	$Atg7^{f/f}$	0	0	0	0	0	0	0	0	0	0	0	1	1	2	2	4	4	2	4	5	4	6	7	10	11	9	8	9	8	8	8	10	9	9	7	9	8	8	9	9	10	11	9	8	8	6	7	5	6	4
WT15	$Atg7^{f/f}$	0	0	0	0	0	0	0	0	0	0	0	0	1	1	1	5	4	4	3	3	4	5	6	6	6	6	6	6	6	6	6	6	6	6	6	6	6	6	6	6	6	6	6	6	6	6	6	6	6	6
WT16	$Atg7^{f/f}$	0	0	0	0	0	0	0	0	2	6	7	9	10	7	7	8	10	9	10	11	9	11	10	10	9	11	8	8	8	8	8	8	8	8	8	8	8	8	8	8	8	8	8	8	8	8	8	8	8	8
WT17	$Atg7^{f/f}$	0	0	0	0	0	0	0	0	0	5	7	3	6	7	6	6	7	7	10	9	8	9	9	12	12	8	11	6	5	5	4	4	4	5	5	6	4	4	4	4	4	4	4	4	4	4	4	4	4	4
WT18	$Atg7^{f/f}$	0	0	0	0	0	0	0	0	1	3	2	2	1	3	2	2	2	3	2	3	3	4	3	4	4	4	2	2	3	3	3	3	3	3	3	3	1	1	2	1	2	1	2	1	1	2	2	2	2	2
WT19	$Atg7^{f/f}$	0	0	0	0	0	0	2	2	2	2	2	2	4	6	6	6	7	7	7	7	6	7	8	7	5	5	4	1	1	1	1	1	1	1	1	1	1	1	1	1	1	1	1	1	1	1	1	1	1	1
WT20	$Atg7^{f/f}$	0	0	0	0	0	0	2	3	3	7	5	6	4	6	5	6	7	7	7	8	10	7	9	8	8	8	4	6	5	4	6	5	4	4	4	4	3	3	3	3	3	3	3	3	3	3	3	3	3	3
EKO1	$Atg7^{\Delta ep}$	0	0	0	0	0	0	0	0	0	1	1	2	2	2	2	2	4	3	2	2	2	2	2	2	2	2	2	2	2	2	2	2	2	2	2	2	2	2	2	2	2	2	2	2	2	2	2	2	2	2
EKO2	$Atg7^{\Delta ep}$	0	0	0	0	0	0	0	1	6	6	3	6	6	8	9	11	8	7	4	3	3	3	3	3	3	1	0	0	0	0	0	0	0	0	0	0	0	0	0	0	0	0	0	0	0	0	0	0	0	0
EKO3	$Atg7^{\Delta ep}$	0	0	0	0	0	0	0	0	0	0	0	0	6	3	3	6	5	9	5	5	4	1	1	1	2	1	1	0	1	1	1	1	1	1	1	1	1	1	1	1	1	1	1	1	1	1	1	1	1	1
EKO4	$Atg7^{\Delta ep}$	0	0	0	0	0	0	0	0	0	0	0	0	0	0	0	0	2	0	1	1	1	0	1	0	0	3	4	1	1	1	1	1	1	0	0	0	0	0	0	0	0	0	0	0	0	0	0	0	0	0
EKO5	$Atg7^{\Delta ep}$	0	0	0	0	0	0	0	0	0	0	1	1	1	2	2	2	3	3	3	6	2	5	4	4	3	6	9	7	7	8	7	6	7	7	5	7	5	4	4	4	4	4	4	1	3	3	3	4	4	4
EKO6	$Atg7^{\Delta ep}$	0	0	0	0	0	0	0	1	1	1	1	1	2	2	4	6	7	2	7	7	5	8	8	7	8	5	5	4	2	3	3	2	3	3	3	3	3	3	3	3	3	3	3	3	3	3	3	3	3	3
EKO7	$Atg7^{\Delta ep}$	0	0	0	0	0	0	0	0	1	3	3	7	6	9	9	10	9	11	6	8	8	7	8	6	6	6	6	5	4	6	4	4	4	5	5	5	5	5	5	5	5	5	5	5	5	5	5	5	5	5
EKO8	$Atg7^{\Delta ep}$	0	0	0	0	0	0	0	0	0	0	0	3	3	3	3	1	1	0	2	0	0	0	0	0	2	2	0	2	1	1	1	1	1	1	1	1	1	1	1	1	1	1	1	1	1	1	1	1	1	1
EKO9	$Atg7^{\Delta ep}$	0	0	0	0	0	0	0	0	5	5	5	2	2	2	7	7	2	4	4	2	2	1	2	1	1	1	3	3	3	2	2	2	2	2	2	1	1	1	1	1	1	1	0	1	1	1	1	1	1	1
EKO10	$Atg7^{\Delta ep}$	0	0	0	0	0	0	0	0	0	0	4	6	9	7	8	16	9	6	11	8	6	8	7	9	5	4	2	1	2	2	2	2	2	2	2	2	2	1	1	1	1	1	1	1	1	1	1	1	1	1
EKO11	$Atg7^{\Delta ep}$	0	0	0	0	0	0	0	0	1	1	2	2	4	4	4	5	5	4	6	6	5	4	4	6	4	5	5	3	2	4	0	0	2	2	2	2	2	2	2	2	2	2	2	2	2	2	2	2	2	2
EKO12	$Atg7^{\Delta ep}$	0	0	0	0	0	0	0	0	1	5	5	7	10	3	3	4	3	3	3	4	4	4	3	2	1	2	2	1	2	0	0	0	0	0	0	0	0	0	0	0	0	0	0	0	0	0	0	0	0	0
EKO13	$Atg7^{\Delta ep}$	0	0	0	0	0	0	0	2	1	1	4	5	2	2	2	4	2	6	8	8	4	5	5	8	4	7	4	3	2	2	2	2	2	2	3	2	2	2	2	1	1	1	2	2	2	2	2	2	2	2
EKO14	$Atg7^{\Delta ep}$	0	0	0	0	0	1	3	5	5	9	7	5	6	7	3	1	4	1	4	5	3	3	2	2	1	1	0	0	0	0	0	0	0	0	0	0	0	0	0	0	0	0	0	0	0	0	0	0	0	0
EKO15	$Atg7^{\Delta ep}$	0	0	0	0	0	0	0	0	1	1	0	1	2	1	1	2	2	2	1	2	1	0	0	0	0	0	0	0	0	0	0	0	0	0	0	0	0	0	0	0	0	0	0	0	0	0	0	0	0	0
EKO16	$Atg7^{\Delta ep}$	0	0	0	0	0	0	4	3	5	4	7	4	5	6	3	4	6	5	4	9	8	8	8	8	8	8	8	8	8	8	8	8	8	8	8	8	8	8	8	8	8	8	8	8	8	8	8	8	8	8
EKO17	$Atg7^{\Delta ep}$	0	0	0	0	0	0	0	4	2	3	4	4	2	1	4	3	7	6	7	5	8	5	5	5	5	2	2	2	1	1	1	1	1	1	1	1	1	1	1	1	1	1	1	1	1	1	1	1	1	1

Legend: The table shows tumor numbers per mouse. After death the last tumor count is maintained until the end of the study (week 50).

Shading: yellow, tumor-free survival corresponding to timepoints before appearance of the first tumor(s); green, timepoints after disappearance of all previously detected tumors if neither new tumors formed nor death occurred until week 50; blue, dead.

Table S2. Tumor volume of *K5-SOS EGFR*$^{wa2/wa2}$ *Atg7*$^{f/f}$ and *K5-SOS EGFR*$^{wa2/wa2}$ *Atg7*$^{\Delta ep}$ mice after wounding on both ears, including volumes after death of affected mice

Mouse	Genotype	Tumor volume (mm^3) at different times after tumor initiation by wounding (w, week)								
		w4	w5	w6	w7	w8	w9	w10	w11	w12
WTE1	*K5-SOS EGFR*$^{wa2/wa2}$ *Atg7*$^{f/f}$	5	7	19	41	35	39	63	78	65
WTE2	*K5-SOS EGFR*$^{wa2/wa2}$ *Atg7*$^{f/f}$	3	5	10	9	14	35	47	57	104
WTE3	*K5-SOS EGFR*$^{wa2/wa2}$ *Atg7*$^{f/f}$	28	79	182	227	337	435	435	590	977
WTE4	*K5-SOS EGFR*$^{wa2/wa2}$ *Atg7*$^{f/f}$	17	62	123	187	315	424	593	593	593
WTE5	*K5-SOS EGFR*$^{wa2/wa2}$ *Atg7*$^{f/f}$	28	114	217	426	426	426	426	426	426
WTE6	*K5-SOS EGFR*$^{wa2/wa2}$ *Atg7*$^{f/f}$	7	15	36	45	53	273	347	347	347
WTE7	*K5-SOS EGFR*$^{wa2/wa2}$ *Atg7*$^{f/f}$	12	51	149	199	383	587	587	587	587
WTE8	*K5-SOS EGFR*$^{wa2/wa2}$ *Atg7*$^{f/f}$	93	273	564	564	564	564	564	564	564
WTE9	*K5-SOS EGFR*$^{wa2/wa2}$ *Atg7*$^{f/f}$	26	242	371	371	371	371	371	371	371
WTE10	*K5-SOS EGFR*$^{wa2/wa2}$ *Atg7*$^{f/f}$	17	87	220	283	411	586	586	586	586
WTE11	*K5-SOS EGFR*$^{wa2/wa2}$ *Atg7*$^{f/f}$	19	122	249	361	361	231	454	601	502
EKOE1	*K5-SOS EGFR*$^{wa2/wa2}$ *Atg7*$^{\Delta ep}$	21	47	87	114	99	150	177	185	374
EKOE2	*K5-SOS EGFR*$^{wa2/wa2}$ *Atg7*$^{\Delta ep}$	2	3	6	12	18	52	50	59	88
EKOE3	*K5-SOS EGFR*$^{wa2/wa2}$ *Atg7*$^{\Delta ep}$	4	3	7	7	11	17	38	52	57
EKOE4	*K5-SOS EGFR*$^{wa2/wa2}$ *Atg7*$^{\Delta ep}$	15	15	61	22	25	22	25	23	45
EKOE5	*K5-SOS EGFR*$^{wa2/wa2}$ *Atg7*$^{\Delta ep}$	2	4	2	2	1	1	1	0	0
EKOE6	*K5-SOS EGFR*$^{wa2/wa2}$ *Atg7*$^{\Delta ep}$	4	6	13	33	56	136	151	346	426
EKOE7	*K5-SOS EGFR*$^{wa2/wa2}$ *Atg7*$^{\Delta ep}$	4	1	1	2	2	3	5	8	14
EKOE8	*K5-SOS EGFR*$^{wa2/wa2}$ *Atg7*$^{\Delta ep}$	10	22	53	77	143	208	258	233	246
EKOE9	*K5-SOS EGFR*$^{wa2/wa2}$ *Atg7*$^{\Delta ep}$	0	0	0	0	0	0	0	0	0
EKOE10	*K5-SOS EGFR*$^{wa2/wa2}$ *Atg7*$^{\Delta ep}$	0	0	0	0	0	0	0	0	0

Legend: The table shows tumor volumes per mouse. After death the last tumor volume is maintained until the end of the study (week 12 after wounding).
Shading: blue, dead.

Table S3. Tumor volume on each ear of *K5-SOS EGFR*$^{wa2/wa2}$ *Atg7*$^{f/f}$ and *K5-SOS EGFR*$^{wa2/wa2}$ *Atg7*$^{\Delta ep}$ mice

Mouse	Genotype	Ear	Tumor volume (mm^3) at different times after tumor initiation by wounding (w, week)								
			w4	w5	w6	w7	w8	w9	w10	w11	w12
WTE1	*K5-SOS EGFR*$^{wa2/wa2}$ *Atg7*$^{f/f}$	R	4	4	13	**38**	**34**	**38**	**62**	**77**	**64**
WTE2	*K5-SOS EGFR*$^{wa2/wa2}$ *Atg7*$^{f/f}$	R	2	4	8	8	13	**34**	**46**	**56**	**103**
WTE3	*K5-SOS EGFR*$^{wa2/wa2}$ *Atg7*$^{f/f}$	R	18	**47**	**109**	**116**	**151**	**173**	**199**	**332**	**349**
WTE4	*K5-SOS EGFR*$^{wa2/wa2}$ *Atg7*$^{f/f}$	R	8	**47**	**65**	**111**	**195**	**219**	**295**	**295**	**295**
WTE5	*K5-SOS EGFR*$^{wa2/wa2}$ *Atg7*$^{f/f}$	R	19	**62**	**151**	**324**	**324**	**324**	**324**	**324**	**324**
WTE6	*K5-SOS EGFR*$^{wa2/wa2}$ *Atg7*$^{f/f}$	R	6	14	**33**	**40**	**42**	**259**	**325**	**325**	**325**
WTE7	*K5-SOS EGFR*$^{wa2/wa2}$ *Atg7*$^{f/f}$	R	8	**38**	**109**	**139**	**239**	**344**	**344**	**344**	**344**
WTE8	*K5-SOS EGFR*$^{wa2/wa2}$ *Atg7*$^{f/f}$	R	**43**	**155**	**295**	**295**	**295**	**295**	**295**	**295**	**295**
WTE9	*K5-SOS EGFR*$^{wa2/wa2}$ *Atg7*$^{f/f}$	R	18	**150**	**217**	**217**	**217**	**217**	**217**	**217**	**217**
WTE10	*K5-SOS EGFR*$^{wa2/wa2}$ *Atg7*$^{f/f}$	R	14	**54**	**135**	**151**	**257**	**416**	**416**	**416**	**416**
WTE11	*K5-SOS EGFR*$^{wa2/wa2}$ *Atg7*$^{f/f}$	R	8	**69**	**146**	**249**	**210**	**118**	**278**	**364**	**335**
WTE1	*K5-SOS EGFR*$^{wa2/wa2}$ *Atg7*$^{f/f}$	L	1	2	6	4	1	2	1	1	1
WTE2	*K5-SOS EGFR*$^{wa2/wa2}$ *Atg7*$^{f/f}$	L	1	1	2	1	1	1	1	1	1
WTE3	*K5-SOS EGFR*$^{wa2/wa2}$ *Atg7*$^{f/f}$	L	9	**33**	**73**	**111**	**187**	**261**	**236**	**258**	**628**
WTE4	*K5-SOS EGFR*$^{wa2/wa2}$ *Atg7*$^{f/f}$	L	8	15	**59**	**75**	**120**	**205**	**299**	**299**	**299**
WTE5	*K5-SOS EGFR*$^{wa2/wa2}$ *Atg7*$^{f/f}$	L	9	**52**	**66**	**102**	**102**	**102**	**102**	**102**	**102**
WTE6	*K5-SOS EGFR*$^{wa2/wa2}$ *Atg7*$^{f/f}$	L	1	1	3	5	11	14	**22**	**22**	**22**
WTE7	*K5-SOS EGFR*$^{wa2/wa2}$ *Atg7*$^{f/f}$	L	4	13	**40**	**60**	**144**	**244**	**244**	**244**	**244**
WTE8	*K5-SOS EGFR*$^{wa2/wa2}$ *Atg7*$^{f/f}$	L	**50**	**118**	**269**	**269**	**269**	**269**	**269**	**269**	**269**
WTE9	*K5-SOS EGFR*$^{wa2/wa2}$ *Atg7*$^{f/f}$	L	8	**92**	**155**	**155**	**155**	**155**	**155**	**155**	**155**
WTE10	*K5-SOS EGFR*$^{wa2/wa2}$ *Atg7*$^{f/f}$	L	4	**32**	**85**	**132**	**155**	**170**	**170**	**170**	**170**
WTE11	*K5-SOS EGFR*$^{wa2/wa2}$ *Atg7*$^{f/f}$	L	11	**53**	**103**	**111**	**151**	**113**	**177**	**237**	**167**
EKOE1	*K5-SOS EGFR*$^{wa2/wa2}$ *Atg7*$^{\Delta ep}$	R	18	**43**	**79**	**98**	**77**	**86**	**86**	**89**	**174**
EKOE2	*K5-SOS EGFR*$^{wa2/wa2}$ *Atg7*$^{\Delta ep}$	R	1	1	3	2	4	4	8	6	6
EKOE3	*K5-SOS EGFR*$^{wa2/wa2}$ *Atg7*$^{\Delta ep}$	R	4	3	6	6	10	17	**38**	**52**	**57**
EKOE4	*K5-SOS EGFR*$^{wa2/wa2}$ *Atg7*$^{\Delta ep}$	R	7	7	**48**	15	18	19	**22**	20	**42**
EKOE5	*K5-SOS EGFR*$^{wa2/wa2}$ *Atg7*$^{\Delta ep}$	R	1	3	1	1	1	1	1	0	0
EKOE6	*K5-SOS EGFR*$^{wa2/wa2}$ *Atg7*$^{\Delta ep}$	R	1	4	11	**29**	**53**	**128**	**141**	**329**	**378**
EKOE7	*K5-SOS EGFR*$^{wa2/wa2}$ *Atg7*$^{\Delta ep}$	R	1	1	1	1	1	1	0	0	0
EKOE8	*K5-SOS EGFR*$^{wa2/wa2}$ *Atg7*$^{\Delta ep}$	R	7	16	**39**	**51**	**82**	**113**	**103**	**85**	**92**
EKOE9	*K5-SOS EGFR*$^{wa2/wa2}$ *Atg7*$^{\Delta ep}$	R	0	0	0	0	0	0	0	0	0
EKOE10	*K5-SOS EGFR*$^{wa2/wa2}$ *Atg7*$^{\Delta ep}$	R	0	0	0	0	0	0	0	0	0
EKOE1	*K5-SOS EGFR*$^{wa2/wa2}$ *Atg7*$^{\Delta ep}$	L	2	4	8	15	**23**	**64**	**90**	**97**	**199**
EKOE2	*K5-SOS EGFR*$^{wa2/wa2}$ *Atg7*$^{\Delta ep}$	L	1	2	2	10	15	**48**	**42**	**53**	**82**
EKOE3	*K5-SOS EGFR*$^{wa2/wa2}$ *Atg7*$^{\Delta ep}$	L	1	1	1	1	1	0	0	0	0
EKOE4	*K5-SOS EGFR*$^{wa2/wa2}$ *Atg7*$^{\Delta ep}$	L	8	8	14	6	7	3	2	2	2
EKOE5	*K5-SOS EGFR*$^{wa2/wa2}$ *Atg7*$^{\Delta ep}$	L	1	1	1	0	0	0	0	0	0
EKOE6	*K5-SOS EGFR*$^{wa2/wa2}$ *Atg7*$^{\Delta ep}$	L	2	2	1	4	3	8	9	17	**48**
EKOE7	*K5-SOS EGFR*$^{wa2/wa2}$ *Atg7*$^{\Delta ep}$	L	3	1	1	2	2	2	5	8	14
EKOE8	*K5-SOS EGFR*$^{wa2/wa2}$ *Atg7*$^{\Delta ep}$	L	3	5	14	**26**	**61**	**95**	**155**	**148**	**154**
EKOE9	*K5-SOS EGFR*$^{wa2/wa2}$ *Atg7*$^{\Delta ep}$	L	0	0	0	0	0	0	0	0	0
EKOE10	*K5-SOS EGFR*$^{wa2/wa2}$ *Atg7*$^{\Delta ep}$	L	0	0	0	0	0	0	0	0	0

Legend: The table shows tumor volumes on the right (R) and left (L) ear of each mouse. After death the last tumor volume is maintained until the end of the study.

Bold fonts: tumor > 20 mm^3. Shading: blue, dead.
